# At-Home Breath Data Collection for Signatures of Type 2 Diabetes: A Pilot Clinical Study

**DOI:** 10.3390/bios15030149

**Published:** 2025-02-27

**Authors:** Sokiyna Naser, Deborah A. Roberts, Sudhir Shrestha

**Affiliations:** 1 Intelligent Systems Lab, Sonoma State University, Rohnert Park, CA 94928, USA; nasers@sonoma.edu; 2 Department of Nursing, Sonoma State University, Rohnert Park, CA 94928, USA; robertde@sonoma.edu

**Keywords:** Type 2 diabetes mellitus, blood glucose monitoring, volatile organic compounds, breath diabetes detection, breath analysis, non-invasive biomarkers, at-home data collection

## Abstract

This study investigates the potential of volatile organic compounds (VOCs) in breath as non-invasive biomarkers for monitoring blood glucose levels in individuals with Type 2 diabetes mellitus (T2DM). A pilot clinical study was conducted to explore the correlation between VOCs and blood glucose levels in six T2DM patients. Participants used a custom-developed sensor device to collect breath data at home, alongside finger-stick blood glucose readings. Breath data were transmitted to a cloud database, while blood glucose readings were recorded on paper charts. The sensor data from the device and the blood glucose readings from the charts were consolidated to create the study dataset. Support vector machine and random forest models were employed to analyze the dataset, which achieved accuracies of 85% and 82%, respectively. The results demonstrate the feasibility of at-home breath sensor data collection for clinical studies and suggest its potential as a viable alternative to traditional invasive glucose monitoring methods. Future studies will expand the dataset to include more participants and additional clinical variables to enhance model performance and predictive power. This research highlights the promise of non-invasive breath analysis for glucose monitoring, which could improve patient compliance and diabetes management.

## 1. Introduction

Type 2 diabetes mellitus (T2DM) is an outcome of insulin resistance and is associated with comorbidities such as obesity, hypertension, and dyslipidemia [1]. Type 2 diabetes is a chronic condition that poses severe health risks if unmanaged. Diabetes affects individuals’ functional capacities and quality of life, leading to significant morbidity and premature mortality. Recently, concerns have been raised that more than one-third of diabetes-related deaths occur in people under the age of 60 [2]. There is a 20% higher risk of breast cancer and a two-fold greater risk of developing endometrial and intrahepatic cholangiocarcinoma among adults with T2DM and a high body mass index (BMI) [3]. Traditional monitoring methods, while effective, are often invasive and inconvenient, necessitating the exploration of alternative, non-invasive techniques [3]. For most diabetic populations checking their blood glucose is just a part of life. The two main types are blood glucose meters, which use a drop of blood to check what the levels are at that moment, and continuous glucose monitors (CGMs, which check blood glucose regularly day or night [4]. Non-adherence to the treatment plan is a key factor complicating diabetes management, significantly impacting T2DM health outcomes. Poor medication adherence has been linked to increased costs related to emergency room (ER) visits, outpatient care, hospitalization, and the management of T2DM complications, ultimately contributing to increased mortality [5]. The developments observed from all of these investigations suggest that non-invasive glucose monitoring has a huge potential to supplement the current standard of invasive and minimally invasive approaches with pain-free, efficient, and accurate diabetes management [6]. The lack of effective non-invasive monitoring tools for T2DM available in the market leads to poor management of the condition among patients with Type 2 diabetes who need to use the finger-stick test multiple times a day.

Breath analysis is one of the non-invasive procedures for monitoring diseases that is particularly attractive for patients who have to assess physiological parameters, such as diabetics, who routinely monitor their blood glucose levels [7]. Some volatile organic compounds (VOCs), notably isoprene (heart disease), acetone (diabetes), toluene (lung cancer), nitrogen monoxide (asthma), pentane (heart disease), and ammonia (kidney dysfunction), are established indicators that anticipate underlying disorders [8,9,10]. Sensors attuned to particular VOCs can identify alterations in breath composition. For instance, research demonstrates that an electronic nose can differentiate diabetic from non-diabetic subjects, with up to 90% accuracy [11].

Various methods have been employed for the collection of breath samples in research settings. For instance, Yan et al. (2014) utilized a breath analysis system for diabetes screening where patients were directed to exhale into a 600 mL Tedlar gas bag through a disposable mouthpiece [11]. Alternative methods include the use of gas sample bags, syringes, or rigid containers such as glass or steel without pre-concentrating the samples. Periodically, these samples are stored at freezing temperatures to preserve their integrity, although some degree of deterioration is often inevitable. Despite the versatility and power of established methods like gas chromatography–mass spectrometry (GC-MS), they present significant challenges. The main issue is maintaining the authenticity of the sample from the point of collection through to analysis. Vulnerable compounds may degrade, be altered during storage, or not be collected with adequate selectivity or without losses. Furthermore, both sample collection and analysis are time-intensive processes that require offline handling in the laboratory, complicating the measurement process and increasing costs. Such challenges are particularly acute when results are needed swiftly and in a cost-effective manner. The limitations of current controlled and sanitized breath data collection methods, although effective in laboratory settings, fail to capture the variability and complexity of real-world environments. Additionally, current methods cannot be scaled to collect enough data needed to train complex machine learning algorithms. Thus, the need for this study arises from the lack of authentic, real-time data collection and analysis methods. This paper offers a novel solution by collecting sensor data in real-world scenarios. By training algorithms on data captured from varied environments, the system is better equipped to provide more accurate results in different life settings. This approach enhances the reliability of breath-based glucose monitoring, enabling it to account for the variations of daily activities and environmental factors, ultimately leading to better diabetes management.

In response to these challenges, this research introduces a novel at-home data collection method that captures VOC sample measurements instantly. This approach not only circumvents the logistical and economic burdens associated with traditional methods but also supports more frequent monitoring, representing a substantial advancement in the field. The pilot study presented in this paper involved six participants who used a custom sensor device to collect breath sensor data alongside finger-stick blood glucose readings. The data were analyzed using support vector machine (SVM) and random forest models, which achieved accuracies of 85% and 82%, respectively. This study offers a practical alternative to invasive glucose monitoring, demonstrating the potential of breath analysis in real-world settings for better diabetes management through increased compliance. The materials and methods, including the device, patient recruitment, and at-home data collection, are presented in Section 2. The results, including dataset analysis, correlations between sensor readings and blood glucose levels, and the performance of the machine learning models, are discussed in Section 3. Section 4 provides a discussion on model performance, comparing SVM and random forest models and highlighting areas for future improvements.

## 2. Materials and Methods

**Figure 1:**
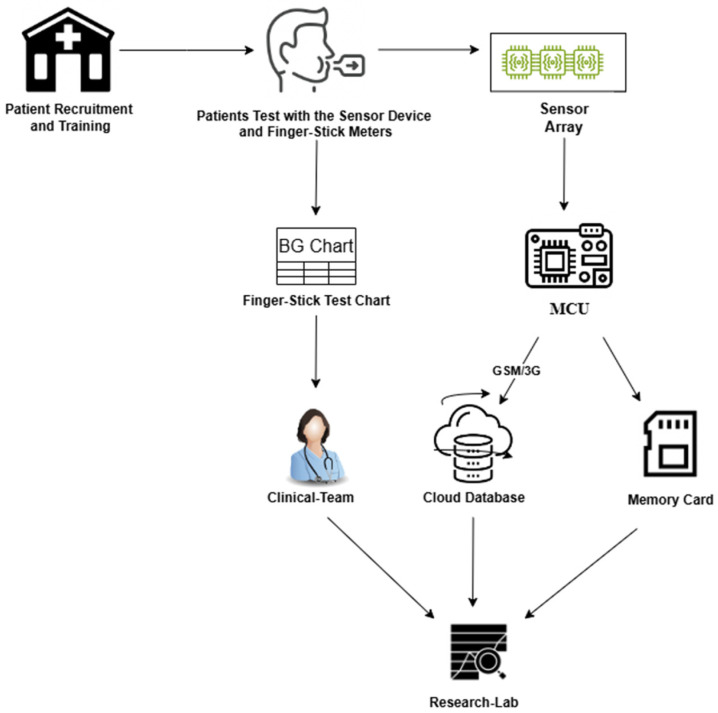
A high-level schematic of the methods and key materials and steps used in this study.

At-home self-testing by patients is the key characteristic and differentiator from similar previously conducted studies. The at-home self-test by the patient allows for the collection of breath data in a real-world scenario, which is necessary to develop solutions that can perform well in real-world scenarios. Figure 1 shows a high-level presentation of methods and materials used in this study. Patients were recruited and informed about the study at a local clinic by a healthcare professional team (clinical team). They were also provided with a finger-stick test chart sheet (BG chart) to record blood glucose readings, as well as necessary information and documentation. Patients were trained to use a hand-held sensor and sent home with the device. Patients who conducted one or more finger-stick tests a day were recruited. Each time the patients conducted a finger-stick test as recommended by their healthcare providers, they also tested with the sensor device and recorded their results on the finger-stick test chart sheet. Patients were asked to breathe into the device twice following the device test prompts. The sensor device recorded sensor readings prior to a patient blowing into the device (sensor array) and while the patient was actively blowing into the sensor. The sensor data were transmitted to a cloud database and were also stored on an onboard memory card as a backup. At the conclusion of the study, the patients returned the sensor device and the BG chart back to the clinical team for de-identification and delivery to the PI’s lab (research lab). The data from the BG chart, memory card, and cloud database were consolidated to create the study dataset.

### 2.1. Clinical Team, Patient Recruitment, and Training

**Figure 2:**
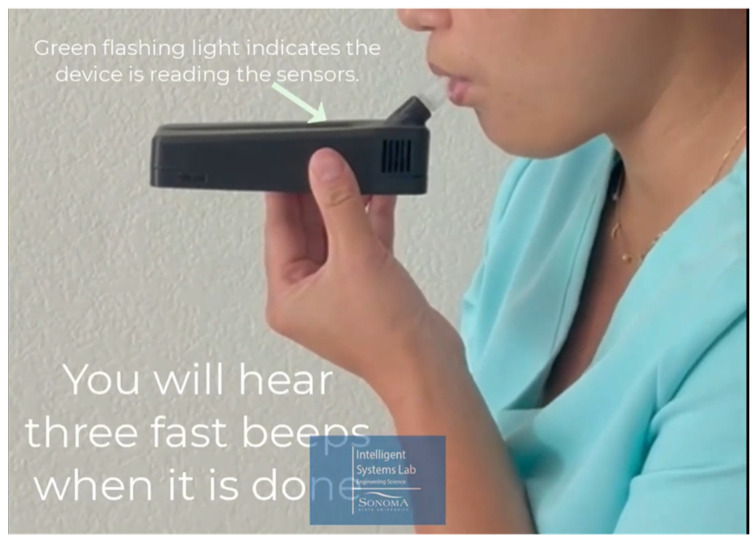
Snapshot of a video used by the clinical team to train the patients on how to use the sensor device.

The clinical team, consisting of a Nurse practitioner and two nursing students, recruited six T2DM patients from a clinic in Santa Rosa, CA. The inclusion criteria for participants were as follows: Adults who were 18 or older, had an existing condition of Type 2 diabetes, and were recommended to test with a finger-stick glucometer one or more times. Gender and ethnicity were noted but were not a factor for recruitment. The device ID numbers were noted before handing the device to patients. The exclusion criteria were Type 1 diabetes patients, patients who used continuous glucose meters (GCMs), and patients who did not need daily finger-stick testing. During their regular clinic visits, patients were trained by the clinical team on how to use the sensor device, after which they signed the informed consent form and were given the device to take home for use alongside their regular glucometer or one provided by the clinic. Patients were informed about the procedure using an information sheet and performing a demonstration on operating the device and breathing into the sensor’s chamber. A snapshot of the demonsration process is shown in Figure 2. This study did not collect any patient-identifying information, and all data were de-identified before sending them to the research lab. A light-emitting diode (LED) indicator served as a guide for patients in using the device and helped to avoid misinterpretation when there was no medically relevant information derived from the device for the patients. The test devices were not reused or retained. Patients were instructed to skip the test with the sensor device if they had any medical emergency or if they perceived any risks. Participants received a new device, which was cleaned before sending them to the clinic, and necessary supplies to complete regular finger sticks and monitoring. The research lab developed a protocol for cleaning the devices. The sensors in the device were located in a chamber isolated from the rest of the electronics. The chamber was cleaned by running ultra-pure air. Upon return of the devices from patients, they were discarded after resetting and deleting all digital information.

### 2.2. Sensor Device

**Figure 3:**
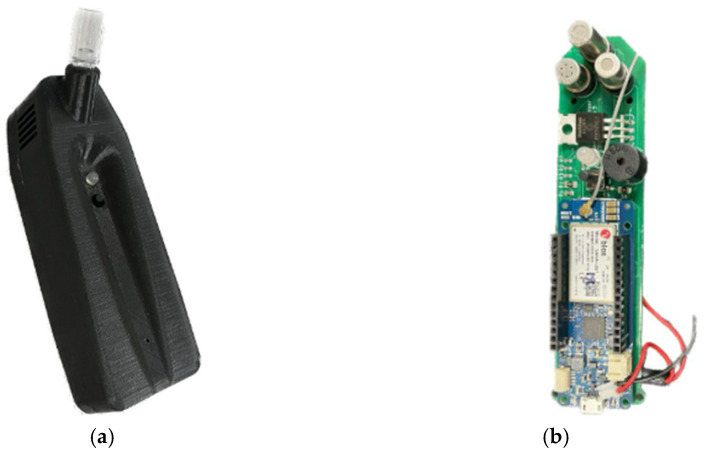
The sensor device developed at the Intelligent Systems Lab, Sonoma State University, which was used in this study: (**a**) packaged sensor device showing the mouthpiece at the top, the LED indicator, and the button. The charging port is located at the bottom; (**b**) the printed circuit board (PCB) of the device showing three VOC sensors at the top and the microcontroller at the bottom. The battery and the antenna are not shown.

A small hand-held device with a VOC sensor array was used to conduct the pilot clinical study presented here (Figure 3). The device was designed and fabricated at the Intelligent Systems Lab (the research lab) at Sonoma State University [12]. The exterior of the device is approximately 1″ × 1.5″ × 6″. The device includes an array comprising three metal oxide semiconductor (MOS)-based VOC sensors, a 32-bit low-power Arm Cortex microcontroller unit (MCU), a global system for mobile/third generation (GSM/3G) chip that supports multiple cellular bands, a rechargeable lithium polymer battery, a charging module, a printed circuit board (PCB) antenna, and a sensor interface circuitry. The MCU has seven analog-to-digital converters (ADCs) and eight digital input/output (I/O) pins. A subscriber identification module (SIM) card allows for sending data using a cellular network. A multicolor LED guides the user through the testing process. A 5-volt USB adapter was used to charge the device.

The sensor device consisted of an array of three VOC sensors. VOCs are emitted as metabolic byproducts, and their concentrations can vary due to several factors, including health conditions. Sensors designed to detect specific VOCs in breath samples can identify these variations, offering a potential early indication of disease. In this study, MOS-based chemical sensors TGS 2620 [13], TGS2602 [14], and TGS 2612 [15] were utilized within the sensing device. The sensors on the array were sensitive to a wide range of VOCs, including acetone, ethanol, methane, iso-butane, hydrogen, ammonia, and hydrogen sulfide, with a sensitivity ranging from 1 to 100 s of ppm. The sensors were selected based on our prior experience working with them, during which we conducted a systematic study comparing two specific VOCs, namely acetone and ethanol, both known to correlate with blood glucose levels [I]. We also tested the sensors with simulated breath containing low and high blood glucose levels, as reported in [16]. Our selection of these sensors and the VOCs for testing was informed by our prior studies reported in [17,18], as well as a literature review of similar studies. We conducted a stability test for the selected sensors by creating a 3 × 3 sensor array (three copies of each sensor) and testing at 10 min intervals. Measured as the percentage deviation from the initial reading, all three sensors remained within 5% of the initial reading for the first 100 cycles. This provided sufficient stability for the presented study, which included a maximum of 90 test cycles.

All three sensors were resistor-based models. We designed a voltage divider circuit and measured the voltage changes using the analog input of a microcontroller with a 10-bit analog-to-digital converter (ADC). As described in Section 2.3, “At-Home Patient Self-Test Procedure”, after starting the device, patients wait for a “breathe-in” prompt. During this time, the sensors are heated through a built-in heater in the sensors. We captured the early response signals of the sensors for this study. Each device was tested to ensure that all sensors and the rest of the electronics performed as expected.

### 2.3. At-Home Patient Self-Test Procedure

Patients were instructed to test with the sensor device each time they conducted a finger-stick test. Right after completing the finger-stick test, patients recorded their finger-stick glucose reading on the BG chart provided to them, along with the date and time of the test (timestamp). After the sensor device started, upon receiving a prompt from the device to blow into the sensor, patients blew into the sensor chamber through the mouthpiece. This process was repeated twice following the prompts from the device. Each patient tested with the device as instructed for approximately three months and returned the device to the clinical team at the end of the study period.

### 2.4. Data Acquisition and Processing

Three sets of data were simultaneously recorded for each test: finger-stick blood glucose levels on the BG chart, sensor array data in the local memory card of the device, and the sensor array data in the database in the cloud (via automatic wireless transmission). In addition, each data point was sent three times for redundancy, which explains why there were a total of 170 data points. Upon the completion of the study period for each patient, the clinical team collected the sensor device and the BG chart, removed all patient-identifying information, and delivered them to the research lab for analysis. The unique device IDs on the BG chart, the memory card, and the cloud database, as well as the timestamp of each test, were used to consolidate the data and create a dataset for further analysis.

## 3. Results

### 3.1. Dataset

**Figure 4:**
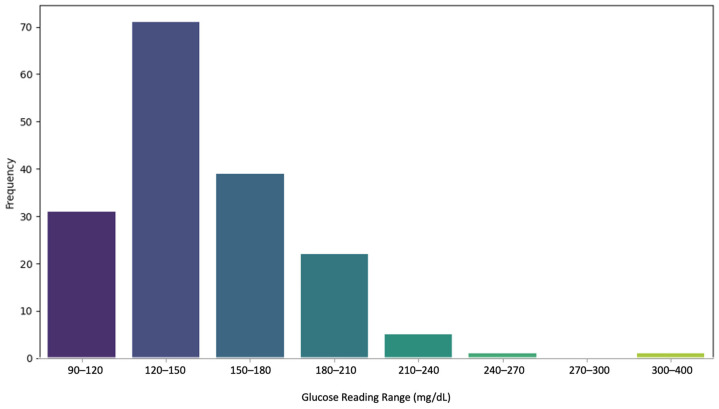
The distribution of finger-stick glucose readings in the dataset.

A total of 744 observations were made after consolidating cloud databases and readings retrieved from the Secure Digital (SD) memory cards on the sensor devices that were returned to the research lab. Each data point sent by or saved on the patients’ devices was timestamped using the Epoch time formula for reliable data synchronization. The finger-stick BG chart readings received from the patients were mapped to the sensor readings’ record using the date and time on the BG chart and timestamps on the sensor readings. We encountered instances of missing data on the patient sheets, which were presented in two scenarios. Either the device reading was recorded, but the patient did not record that on the sheet, or the data were not recorded, but the patient logged a BS on their finger-stick log. Finger-stick test readings were between 96 mg/dL and 393 mg/dL. Figure 4 shows the distribution of the BG readings in various BG ranges.

### 3.2. The Distribution of Finger-Stick Glucose Readings in the Dataset

**Figure 5:**
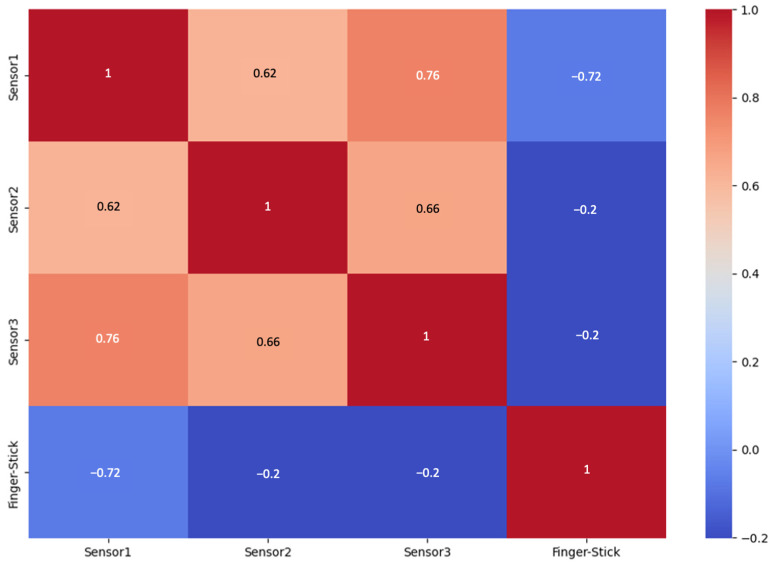
Correlation heatmap of finger-stick BG tests and individual sensors of the VOC sensor array.

The heatmap shown in Figure 5 illustrates the correlation between our target variable, the finger-stick test values, and the features, represented here by the VOC sensor readings in our dataset. A correlation of 1 indicates a strong positive correlation, meaning that as finger-stick test values increase, sensor readings also increase. Conversely, a correlation of −1 indicates a strong negative correlation, i.e., an increase in finger-stick test values corresponds to a decrease in sensor readings. On the heatmap, the diagonal from the bottom left to the top right corner shows the correlation of each variable with itself. Notably, Sensor 3 and Sensor 2 have a −0.20 correlation with the finger-stick test, indicating a modest negative correlation. Sensor 1, however, displays a weak negative correlation with the finger-stick test. While common practice in feature engineering might suggest excluding features with weak correlations for machine learning models, we included all sensor readings in our analysis. This approach allowed us to explore all features thoroughly. The −0.20 correlation suggests that the relationship between these sensors and the finger-stick test does not follow a simple linear pattern, thus necessitating further investigation to uncover more complex functional relationships within the data.

### 3.3. Classification of Finger-Stick BG Tests

**Figure 6:**
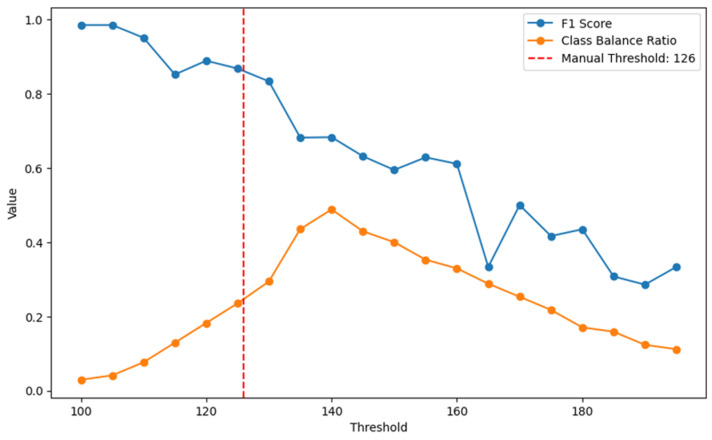
Optimization of classification threshold: plots of F1 score and class balance ratio. The red vertical line depicts the manual threshold at 126 mg/dL.

In our analysis, we aimed to classify subjects into two classes based on finger-stick BG test values. We employed two key metrics: the F1 score and class balance ratio [19]. These metrics were plotted as functions of varying threshold values, as shown in Figure 6. The F1 score (blue line) combines precision and recall into a single measure, capturing the accuracy of our classification at different thresholds. It is particularly useful in scenarios where class imbalance might affect the accuracy of our model. As the threshold increases, the F1 score generally decreases, indicating that raising the threshold might lead to poorer classification performance in terms of both precision and recall. The class balance ratio (orange line) measures the balance between the two classes defined by the threshold. A higher ratio indicates a more equal distribution between the two classes, which is desirable to ensure that the model performs uniformly across different categories. As observed from the chart, there is a notable interaction between the F1 score and the class balance ratio around the threshold values of 125 to 130 mg/dL. In this range, the F1 score is relatively high, and the class balance ratio also reaches a peak, suggesting a good compromise between classification accuracy and class distribution. Based on these observations, we selected 126 mg/dL as the optimal threshold. This threshold was chosen because it represents the best compromise between the F1 score and the class balance ratio. While thresholds beyond 126 mg/dL offered a slightly better class balance ratio, they resulted in a decline in the F1 score, indicating reduced classification performance. Conversely, thresholds below 126 mg/dL maintained higher F1 scores but compromised the class balance ratio. Therefore, 126 mg/dL was identified as the point where the F1 score remained sufficiently high, and the class balance ratio was close to its maximum, ensuring that the model was both accurate and fair in classifying subjects. This balance between accuracy and equity made 126 mg/dL the most effective choice for the threshold.

## 4. Analysis and Discussion

### 4.1. Development of Machine Learning Models

Machine learning (ML) classification techniques have been employed in scientific studies for the diagnosis and prediction of a wide array of diseases and their symptoms [20,21]. For example, the authors of [22] compared several supervised machine learning algorithms for their effectiveness in disease prediction, illustrating the application of techniques like support vector machine (SVM), random forest, and logistic regression models in the healthcare domain. A comparative study on liver disease prediction using supervised machine learning algorithms explored the use of various ML algorithms for predicting liver disease, demonstrating the potential to lower diagnosis costs and improve predictive accuracy [23]. In addition, the connection between sensor data and glucose levels lacks a direct correlation with established physical equations. Consequently, researchers have turned to classical machine learning (ML) models. In the development of our machine learning models, our primary objective was to predict the specified classes, Class 0 and Class 1, based on breath samples collected via the sensor device. For this analysis, we specifically narrowed our investigation to two supervised classifiers known for their effectiveness as classifiers: support vector machine (SVM) and random forest (RF).

SVM functions by identifying the optimal hyperplane that divides different classes within the dataset. This hyperplane is selected to enhance the margin, defined as the space between the hyperplane itself and the closest data points from each class, referred to as support vectors [24]. The random forest algorithm constructs numerous decision trees (DTs) by choosing random segments of the initial dataset and random feature subsets for each tree. The development of each DT within the RF framework employs a method called recursive partitioning. This method sequentially divides the data into smaller groups based on the attributes that provide the most significant separation, culminating in a hierarchical tree structure [24].

### 4.2. Model Performance Analysis

We evaluated the performance of the two chosen machine learning models, support vector machine and random forest, using various metrics such as precision, recall, the F1 score, and overall accuracy [25]. The primary dataset consisted of breath samples, categorized into two classes based on finger-stick test values, with the goal of predicting these classes effectively. The results are shown in Table 1.

### 4.3. Analysis Results

#### 4.3.1. Support Vector Machine

The SVM model demonstrated superior capability in class discrimination, especially for Class 1. SVM’s performance, particularly in terms of recall and F1 score for Class 1, highlights its efficacy in dealing with class imbalances and its ability to effectively segregate distinct classes using a hyperplane. This suggests a better alignment of SVM with datasets where a balance between precision and recall is crucial.

#### 4.3.2. Random Forest

The random forest model showed moderate success in classifying the data. The balanced performance in Class 1 indicates that the random forest model was effective in identifying true positives, albeit the model was not as precise as that with Class 0. This can be attributed to the inherent nature of random forest models, which excel in handling heterogeneous datasets but may struggle with smaller sample sizes for specific categories.

#### 4.3.3. Comparative Analysis

The comparative analysis between random forest and SVM underscores significant differences in handling data classification tasks. SVM showed a higher capability to manage class imbalances, as evidenced by its recall rate in Class 1. While both models performed well, SVM outperformed random forest in overall accuracy and precision.

## 5. Discussion

This pilot study explored the classification of subjects into two categories based on finger-stick test values using the F1 score and class balance ratio, with an optimal threshold identified at 126 mg/dL. This threshold was chosen due to its balanced high F1 score and near-maximum class balance ratio, ensuring equitable class representation and enhancing the model’s generalizability. In terms of model performance, both random forest and SVM models showed robust classification capabilities, with SVM slightly outperforming random forest in overall accuracy and recall for Class 1. Despite their theoretical differences, both models produced identical results in the confusion matrix analysis, highlighting their effectiveness under the study conditions and suggesting that both models are capable of capturing key classification patterns effectively.

Future studies will involve a larger sample of individuals with blood glucose levels above 90 mg/dL to improve model accuracy and representation. Additionally, the dataset will be expanded to include key demographic and clinical features such as body mass index (BMI), age, gender, and detailed disease history, which are anticipated to enhance the model’s predictive power and clinical utility. Concurrently, we will conduct rigorous hyperparameter tuning to refine model performance as new data are integrated, ensuring that our models adapt effectively to increased dataset complexity and maintain high predictive accuracy in diverse clinical scenarios. Eventually, this research advances the potential of non-invasive monitoring techniques in diabetes management, leveraging novel sensor technologies and machine learning models to improve patient outcomes and compliance.

## 6. Conclusions

This pilot study successfully demonstrated the potential of leveraging volatile organic compounds (VOCs) in breath as non-invasive biomarkers for monitoring blood glucose levels in individuals with Type 2 diabetes mellitus (T2DM). Using a sensor-based device, we collected breath samples synchronized with finger-sticktests and applied support vector machine (SVM) and random forest models to analyze the data. These models achieved accuracies of 85% and 82%, respectively, validating their effectiveness as viable alternatives to traditional invasive monitoring methods. Moreover, our research highlights the importance of expanding the dataset to improve model performance. Future iterations of the study will include additional clinical variables such as body mass index (BMI), age, and detailed disease history. These enhancements are expected to significantly boost the diagnostic power of the models. As we move forward, efforts will focus on hyperparameter tuning, increasing sample size, and exploring the impact of environmental factors on VOC emissions. This work lays the foundation for a personalized, non-invasive approach to diabetes management that could improve patient outcomes, enhance compliance with monitoring routines, and reduce healthcare costs through early detection and intervention.

## Figures and Tables

**Table 1:** Performance metric comparison of SVM and random forest models.

Metrics	SVM Model	Random Forest Model
Precision	84.22	81.88
Recall	85.33	81.88
F1 Score	84.67	81.88
Number of Instances	36	36
Test Samples’ Percent	20	20
Overall Model Accuracy	85%	82%

## Data Availability

The data presented in this study are available on request from the corresponding author due to privacy reasons.
